# A Blanket Accommodative Sleep Posture Classification System Using an Infrared Depth Camera: A Deep Learning Approach with Synthetic Augmentation of Blanket Conditions

**DOI:** 10.3390/s21165553

**Published:** 2021-08-18

**Authors:** Andy Yiu-Chau Tam, Bryan Pak-Hei So, Tim Tin-Chun Chan, Alyssa Ka-Yan Cheung, Duo Wai-Chi Wong, James Chung-Wai Cheung

**Affiliations:** 1 Department of Biomedical Engineering, Faculty of Engineering, The Hong Kong Polytechnic University, Hong Kong, China; andy-yiu-chau.tam@connect.polyu.hk (A.Y.-C.T.); bryan.so@connect.polyu.hk (B.P.-H.S.); tctimchan@polyu.edu.hk (T.T.-C.C.); 19062643d@connect.polyu.hk (A.K.-Y.C.); 2 Research Institute for Smart Ageing, The Hong Kong Polytechnic University, Hong Kong, China

**Keywords:** sleep posture recognition, convolutional neural network, sleep disorder, sleep behavior, sleep monitoring, sleep surveillance

## Abstract

Surveillance of sleeping posture is essential for bed-ridden patients or individuals at-risk of falling out of bed. Existing sleep posture monitoring and classification systems may not be able to accommodate the covering of a blanket, which represents a barrier to conducting pragmatic studies. The objective of this study was to develop an unobtrusive sleep posture classification that could accommodate the use of a blanket. The system uses an infrared depth camera for data acquisition and a convolutional neural network to classify sleeping postures. We recruited 66 participants (40 men and 26 women) to perform seven major sleeping postures (supine, prone (head left and right), log (left and right) and fetal (left and right)) under four blanket conditions (thick, medium, thin, and no blanket). Data augmentation was conducted by affine transformation and data fusion, generating additional blanket conditions with the original dataset. Coarse-grained (four-posture) and fine-grained (seven-posture) classifiers were trained using two fully connected network layers. For the coarse classification, the log and fetal postures were merged into a side-lying class and the prone class (head left and right) was pooled. The results show a drop of overall F1-score by 8.2% when switching to the fine-grained classifier. In addition, compared to no blanket, a thick blanket reduced the overall F1-scores by 3.5% and 8.9% for the coarse- and fine-grained classifiers, respectively; meanwhile, the lowest performance was seen in classifying the log (right) posture under a thick blanket, with an F1-score of 72.0%. In conclusion, we developed a system that can classify seven types of common sleeping postures under blankets and achieved an F1-score of 88.9%.

## 1. Introduction

Good sleep is imperative to health and well-being [1]. Significant associations have been made between sleep disorders and chronic diseases/conditions such as obesity, diabetes, and hypertension [2,3,4]. Poor sleep or sleep disturbance can contribute to depression, anxiety, and other mental or psychiatric conditions [5]. The prevalence of sleep disorders is 47 individuals among every 1000 in the population, while some reports suggest that half the population may experience sleep disorders [6]. In China, more than one-quarter of adolescents have been reported to suffer from sleep disturbance [7]. Insomnia represents the most common sleep disorder and has been investigated using polysomnography, ballistocardiography, photoplethysmography and actigraphy [1].

Apart from sleep quality, sleep posture constitutes another important category in the field [8]. Different lying or recumbent positions in sleep alters spinal loading, intervertebral disc pressure, and muscle activity [9,10], potentially leading to sleep-related musculoskeletal pain and disorders such as neck spasm, back pain and waking symptoms [11,12]. For example, a prolonged non-symmetrical sleeping posture might lead to structural spinal changes and symptoms [12,13]. Moreover, sleep apnea, sleep paralysis, and nocturnal gastroesophageal reflux can also be associated with poor sleeping posture [14,15,16]. Investigating sleeping posture is of paramount importance in order to understand the pathomechanism and design interventions for sleeping disorders.

Surveillance of sleeping posture is essential for bed-ridden patients because of the risk of pressure sores [17]. Appropriate body turning frequency or changes in sleeping posture can relieve the prolonged localization of pressure that leads to tissue ischemia and necrosis [18]. In addition, it is also essential to monitor sleeping posture for individuals at risk of falling out of bed, particularly elderly people with dementia or delirium, or those at the end of life [19]. While pressure mats and infrared fences have been routinely exploited for this purpose, some researchers developed an integrated depth camera and impulse radar system to monitor wandering behavior and alarm caregivers when elderly people get out of bed [20].

Traditionally, measurement of sleep posture relied on videotaping with manual labeling or self-reported assessments, which could be inaccurate [12,21]. Tang et al. [22] reviewed the nonintrusive technology applied for recognition of sleep posture, including visible light, infrared, and depth cameras [23,24], inertia measurement units with wireless connection [25], and radar/radio sensors [1,8,26]. Although efforts were made to utilize and integrate different sensors for better versatility, there were few attempts to recognize and classify sleeping postures accurately [22]. Body load and pressure patterns represent another area of studies dedicated to sleep posture recognition. A pressure-sensing mattress or distributed pressure sensors embedded in the mattress can be used to estimate changes of posture during sleep [27,28,29]. Recently, machine learning techniques, such as deep learning, support vector machine (SVM), k-nearest neighbors (KNN), and a convolutional neural network (CNN), were applied to improve the accuracy of posture classification for both optical and pressures sensing [23,24,30,31,32,33,34,35]. However, a lack of robustness remained the major issue that limited their application in the field [33].

A state-of-the-art review on sleep surveillance technology highlighted that tracking movements and postures through blankets was the primary barrier to achieving accurate noncontact monitoring, which remained unresolved [36]. To this end, we aimed to develop a sleep posture classifier using deep learning models with an infrared depth camera and blankets with different thicknesses and materials. Accordingly, we implemented fine-grained classification of seven postures with regard to head and leg positions [36] to improve the robustness of the classifier, and coarse classification of the four standard postures (supine, prone, left side-lying, and right side-lying) to facilitate comparison with existing studies.

## 2. Materials and Methods

### 2.1. Participant Recruitment

We recruited 66 adults (40 men and 26 women) from the university campus by convenience sampling. The mean age of the participants was 35.7 years (SD: 17.4, range 18 to 72), and their average height was 167 cm (SD: 18 cm) and weight was 63 kg (SD: 12.23 kg). They had no reported severe sleeping deprivation, sleep disorder, musculoskeletal pain, or deformity. The experiment was approved by the Institutional Review Board of the university (reference no.: HSEARS20210127007). All participants signed informed consent after receiving oral and written descriptions of the experimental procedures before the start of the experiment.

### 2.2. Hardware Setup

The sleeping posture data were collected from participants lying on a standard electronic rehabilitation bed that was 196 cm long by 90 cm wide and 55 cm high. A 3D time-of-flight near-infrared red, blue, green (RGB) camera (RealSense D435i depth camera, Intel Corp., Santa Clara, CA, USA) was mounted 1.6 m above the bed surface on a steel camera stand. The bed and the annotated area with a color-coded paper for labeling the posture scene were within the camera coverage. The resolution of the camera was 848 × 480 pixels at a frame rate of 6 fps. Four blanket conditions were used with three types of blankets resembling a thick (8 cm thick), medium (2 cm thick), and thin (0.4 cm thick) blanket. The product names of the blankets were the FJALLARNIKA extra warm duvet, SAFFERO light warm duvet, and VALLKRASSING duvet, purchased from IKEA (Delft, The Netherlands).

### 2.3. Experimental Procedure

**Figure 1:**
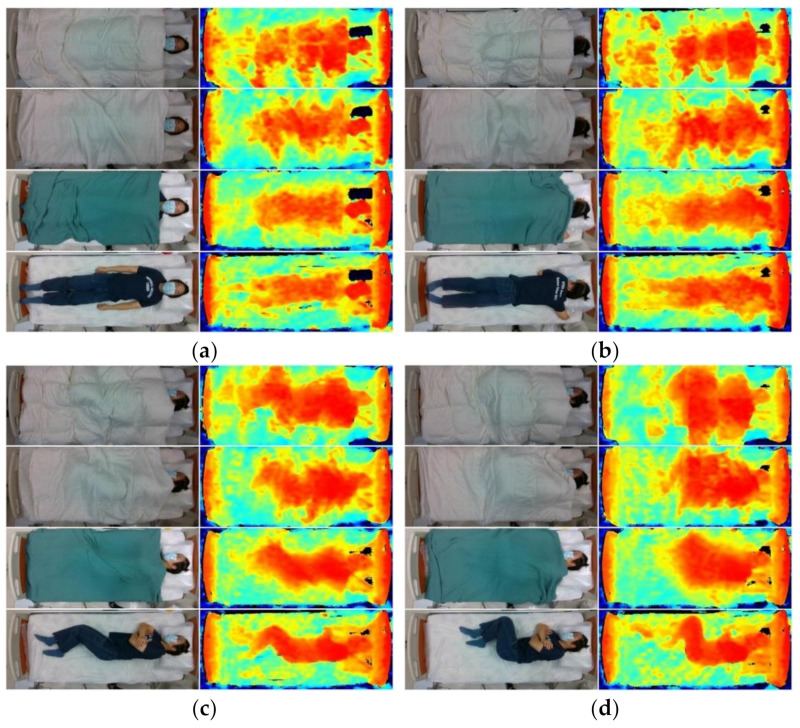
RGB (left column) and infrared (right column) images of typical participant under thick (first row), medium (second row), thin (third row), and control (fourth row) blanket conditions in (**a**) supine, (**b**) prone (head left), (**c**) log (right), and (**d**) fetal (right) postures.

During the experiment, participants were instructed to perform 7 recumbent postures: (1) supine, (2) prone with head turned left, (3) prone with head turned right, (4) log left, (5) log right, (6) fetal left, and (7) fetal right (log and fetal are variations of side-lying). In addition, we instructed the participants to flex their knees at a higher level resembling a cuddle for the fetal position compared to the log position. The supine, prone, log, and fetal positions are demonstrated in Figure 1.

The participants were given time to adjust themselves into their most comfortable position, with a voice cue given for each posture. They were then asked to maintain their position after final adjustment. Each participant was covered with each blanket, from the thickest to no blanket, by the investigators, so that the body position under the different blankets would be identical. The participants were asked to repeat the posture for any observable movement or change in posture without the voice cue. The infrared RGB depth camera continued recording without interruption throughout the entire course of the experiment. For each posture condition, we replaced a color-coded paper next to the bed within the camera coverage to indicate the time for splicing and facilitate labeling and pre-processing of data.

### 2.4. Data Pre-Processing and Augmentation

On the RealSense Software Development Kit (SDK) platform, the RGB and infrared depth image data were aligned and streamlined. Image/video cropping was performed to confine the image to the bed area only through a manual pipeline program on the SDK OpenCV platform. All data were spliced and labeled based on the color-coded paper placed during the experiment and observation. Together with the participant information, the processed and labeled data were organized into a MariaDB database. Out of the 66 participants, the data on 51 were randomly selected for model training, while that of the remaining 15 were used for model testing and evaluation.

The original model training dataset consisted of approximately 1400 data samples (51 participants × 7 postures × 4 blanket conditions). A customized data augmentation strategy was applied to the model training to generate more blanket conditions to account for the limited number of samples. Data augmentation was conducted using affine transformation of the image data and data fusion of the blanket conditions.

The affine transformation was implemented using the Albumentation library, which scaled, translated, rotated, and sheared the images with range shifts of 5%, 2%, 5.0°, and 2.5°, respectively [37]. The range shifts were determined based on empirical observation, such that the participant’s whole body was still covered after image transformation.

For the data fusion, we synthesized an additional dataset based on the original model training dataset and generated more blanket conditions using an intraclass mix-up algorithm with random variables, as shown in Equation (1). The depth images can be interpreted as a linear combination over the multiple positions of the human body and the blanket, under the premise that the participants maintained their posture across blanket conditions during data acquisition. The technique interpolated the depth image data and constructed the hypothetical variations of new blankets. Mathematically, the weighted sum of the two depth images (blanket conditions) was applied, as shown in Equation (1).

(1) $$Fusion\left(img_{1},\;img_{2}\right)=u*img_{1}+\left(1-u\right)\;*\;img_{2}\;,\;where\;u\;∊\;U\left[0,1\right]$$

where *img*_1_ and *img*_2_ are two sets of drawn images, with all combinations, and *u* is a random number drawn from 0 to 1 with uniform distribution.

**Figure 2:**
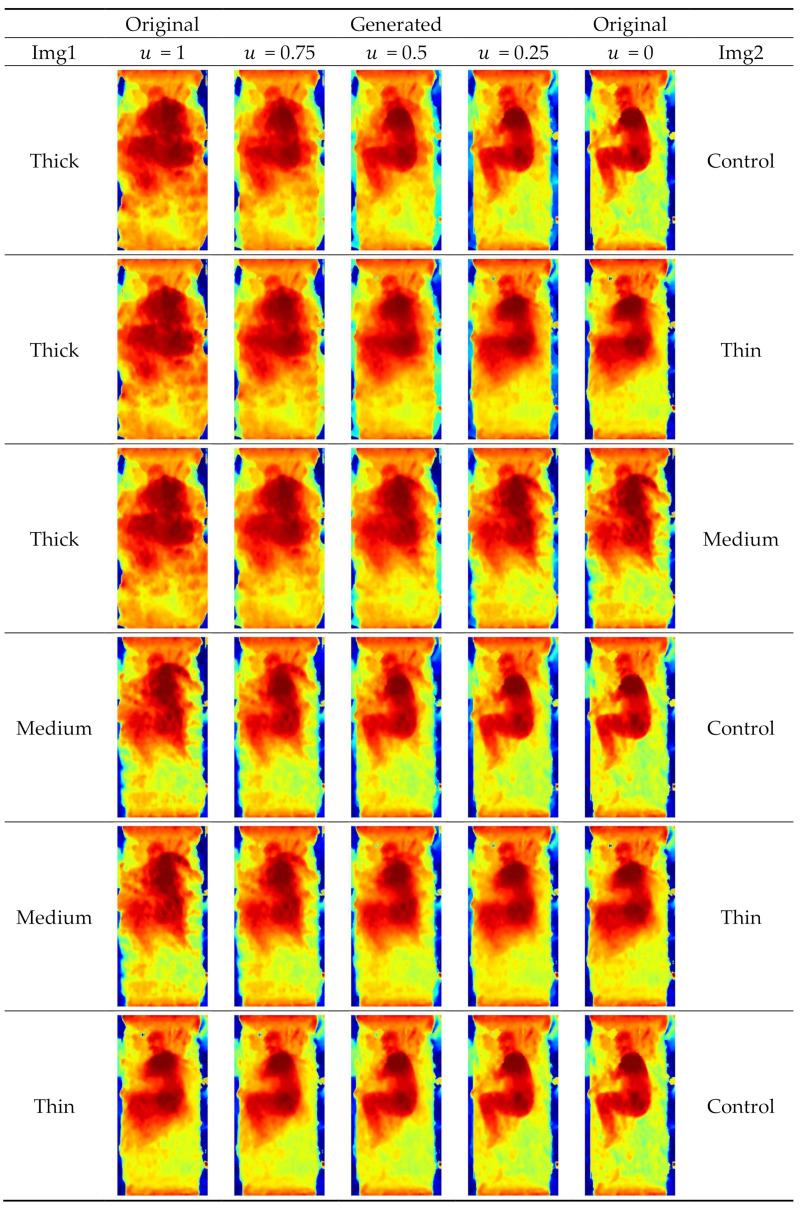
Illustration of data fusion technique on six combinations of blanket conditions.

Four blanket conditions contributed to 6 possible combinations and thus 6 hypothetical blanket conditions for *img*_1_ and *img*_2_ (i.e., the 6 rows in Figure 2). For ease of understanding, we present Figure 2 to illustrate all 6 combinations of blanket pairs with 3 pre-assigned *u* values. It should be noted that a random u value will be assigned in every combination and in every epoch during model training.

Both the original model training dataset (51 participants) and the synthesized dataset were used for the model training. Only the original testing dataset (15 participants) was used for model evaluation and testing.

### 2.5. Model Training and Architecture

**Figure 3:**
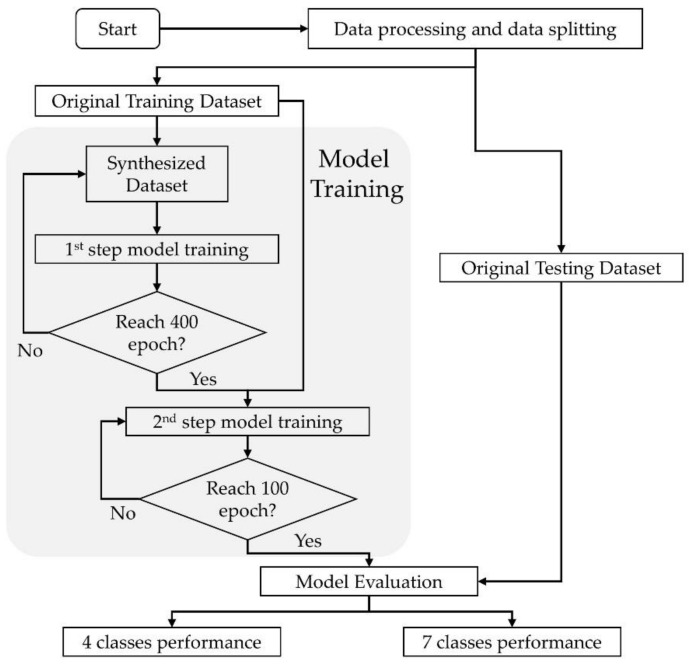
Flowchart of functional component linkage of model network.

**Figure 4:**
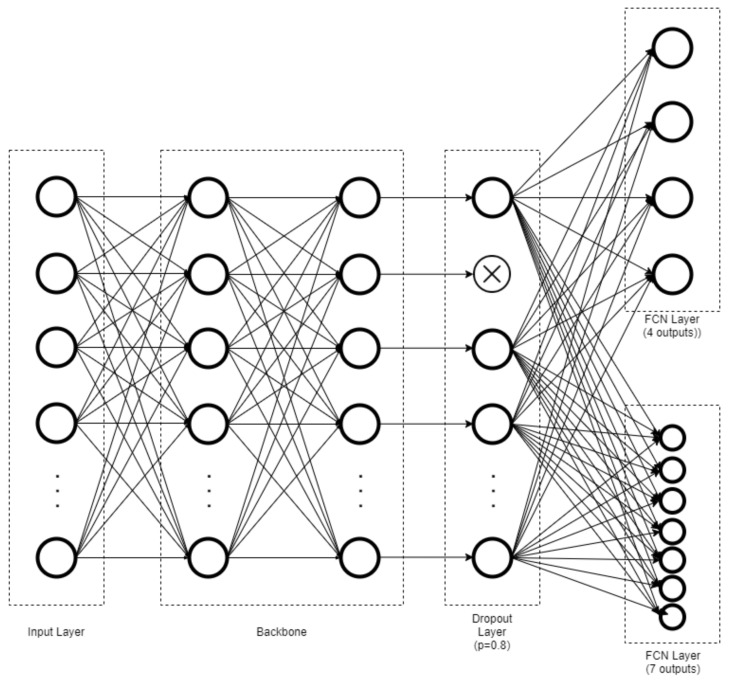
Architecture of model network illustrating fully connected network (FCN) layers toward coarse-grained (4-posture) and fine-grained (7-posture) classification.

A convolution neural network (CNN) with EfficientNet as the backbone [38] over 2 output channels was implemented via TensorFlow Keras v. 2.4.1 (Google Brain Team, Google Alphabet, Mountain View, CA, USA) with Compute Unified Device Architecture, CUDA (Nvidia, Santa Clara, CA, USA). The CNN was built by replacing the last layer of EfficientNet with a dropout layer, followed by 2 parallel fully connected network (FCN) layers, and Softmax was used as the activation function to achieve coarse-(4-posture) and fine-grained (7-posture) classification. The network was initialized with weights pretrained using the “nosy-student” transfer learning method [39]. The dropout layer was assigned with a probability of 0.8 to overcome the overfitting problem. Figure 3 and Figure 4 show flowcharts of the functional component linkage and the architecture of the model network, respectively. The model was subsequently trained using the prepared dataset for the coarse-(4-posture) and fine-grained (7-posture) classification. The fine-grained classification was based on the 7 postures performed by the participants. In the coarse classification, the log and fetal posture classes were merged into the side-lying class, and the prone postures with different head positions were also pooled.

During the model training, the model parameters were determined by stochastic gradient descent to optimize the loss function. The outputs were passed to the categorical cross-entropy loss functions for both the coarse and fine classification, as shown in Equations (2) and (3). Finally, the total loss function was constructed based on a simple sum of losses, as shown in Equation (4):

(2) $$CE_{coarse}\left(y,\;\hat{y}\right)=\sum_{i}^{C=4}y_{i}*\log\left({\hat{y}}_{i}\right)$$

(3) $$CE_{fine}\left(y,\hat{y}\right)=\sum_{i}^{C=7}y_{i}*\log\left(\hat{y_{i}}\right)$$

(4) $$L\left(y,\hat{y}\right)=CE_{coarse}\left(y_{coarse},{\hat{y}}_{coarse}\right)+CE_{fine}\left(y_{fine},{\hat{y}}_{fine}\right)$$

where *C* is the number of classes; $y_{i}$ is an indicator function, which is one if $y_{i}$ belongs to class i and zero otherwise; and $\hat{y_{i}}$ is the predicted probability when the input belongs to class i. $CE_{coarse}\left(y_{coarse},{\hat{y}}_{coarse}\right)$ and $CE_{fine}\left(y_{fine},{\hat{y}}_{fine}\right)$ denote the categorical cross-entropy between the true and predicted output by the network for coarse and fine-grained classifications. The pseudocode of the algorithm is included in the Appendix (Table A1).

The Adam optimizer was used at a fixed learning rate of 0.0001 and L2 regularization of 0.0005. The model was trained in two steps because of the bias in probability density distribution in the synthesized dataset. The model was firstly trained by the synthesized dataset at 400 epochs and afterward trained by the original training dataset at 100 epochs, determined by the observation of the learning curve.

### 2.6. Model Evaluation

The original testing dataset of the 15 participants was used for model evaluation. The model’s performance was evaluated using macro accuracy, recall, precision, and F1 score parameters based on the true/false positives and negatives of the predicted classes. In addition, Cohen’s kappa was used to assess the agreement of the predicted outcome and the dataset labels (ground truth) [40]. These parameters were calculated based on Equations (5)–(9). A confusion matrix across the coarse- and fine-grained classifications was also developed to identify whether the source of errors was mainly the classification technique, posture, or blanket conditions.

(5) $$Recall=\frac{TP}{TP+FN}$$

(6) $$Precision=\frac{TP}{TP+FP}$$

(7) $$Accuracy=\frac{TP+TN}{TP+TN+FP+FN}$$

(8) $$F1\;Score=\frac{2TP}{2TP+FP+FN}$$

Here, *TP*, *FP*, *TN*, and *FN*, are true positive, false positive, true negative, and false negative, respectively.

(9) $$\kappa =\frac{p_{o}-p_{e}}{1-p_{e}}$$

Here, *p_o_* is the empirical probability of agreement of the label assigned to any sample (observed agreement ratio), and *p_e_* is the expected agreement when both annotators assign labels randomly and is estimated using a priori per annotator over class labels [40].

## 3. Results

The overall performance of the coarse- and fine-grained classification is presented in Table 1. In general, the performance of the former was better than that of latter. The accuracy, recall, and precision of the coarse classification model were approximately 8% higher than those of the fine-grained classification. The F1 score of the four-posture classifier was 97.1%, whereas that of the seven-posture classifier was 88.9%. In addition, the four-posture coarse classification model demonstrated excellent reliability, with a Cohen’s kappa of 0.970, whereas the seven-posture fine-grained classification had a Cohen’s kappa coefficient of 0.891, indicating strong reliability between the ground truth set and the predictive set.

A subgroup analysis of the model performance (F1-score) was conducted, stratifying the posture and blanket conditions. The blanket affected the model performance of the classifiers. As seen in Table 2, the overall performance of the coarse- and fine-grained classification declined from 98.6% to 95.1% and from 93.3% to 84.4%, respectively, when the condition shifted from control (no blanket) to thick blanket. The overall F1-score difference between the thin blanket and control was minimal: 0.1% for the coarse-grained and 2% for the fine-grained classification.

Side-lying seemed to produce the best prediction performance in the four-posture coarse classification. The overall F-scores for right and left side-lying were 98.0% and 99.0%, respectively. However, subdividing the side-lying posture into the log and fetal postures affected the model performance. The F1-score for log (right) was 82.8%, which was the lowest among the fine-grained classification scores. The performance was further reduced to 72.0% if only the thick blanket was considered. Moreover, subdividing prone into left and right head positions also weakened the model performance. F1 scores were 75.0% and 81.5% in prone postures with head left and right, respectively, under the thick blanket.

**Figure 5:**
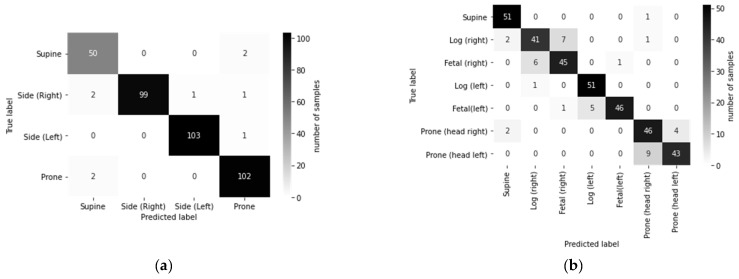
Confusion matrix across true and predicted labels for (**a**) 4-posture coarse-grained classification and (**b**) 7-posture fine-grained classification.

The confusion matrix in Figure 5a shows that the performance in classifying supine and prone was slightly inferior to that of left and right side-lying posture. The confusion matrix in Figure 5b shows that the majority of predicted errors were contributed by the further sub-classification of log and fetal postures and the head facing left and right in prone posture.

## 4. Discussion

The innovation of this study is the robustness provided by including blankets in the classification of sleep postures to enable practical applications. We also classified postures with higher granularity, from the four standard postures (supine, prone, right and left lateral) to seven postures (including head and leg positions) [36]. Another novelty is the use of data fusion techniques to generate various blanket conditions from the original dataset to subsume hypothetical blanket variations to enhance the generalizability of the model. Indeed, the study demonstrates practical value because it is infeasible to apply all available blankets for the investigation. It should be noted that although the results in this paper present both RGB and infrared depth camera images, the deep learning model applied infrared depth images only. Therefore, confidentiality could be compromised in actual applications.

Other posture classification systems have used the depth cameras but with different configurations or levels of classification. Ren et al. [41] developed a system with the Kinect Artec scanner. They classified six postures (without prone) using SVM with the scale-invariant feature transform (SIFT) feature, which yielded an accuracy of 92.5%. The accuracy of our seven-posture fine-grained classifier was higher (93.3%) for the no-blanket condition. Although the overall accuracy of our classifier was lower (89.0%), the experimental design of our dataset allowed for more features, including the prone posture and the interference of blankets. It was challenging to differentiate the supine and prone classes because of the limited depth resolution to trace detailed head/face features. On the other hand, Grimm et al. [42] proposed an alleviation map approach to delineate depth camera images followed by a CNN to classify three postures, supine and left and right side-lying. Their system achieved an accuracy of 94%, while our four-posture coarse classification system performed better with an overall accuracy of 97.5% considering blanket interference.

The performance of the coarse-grained classifier was approximately 8% higher than that of the fine-grained classifier, which could be due to the feature-diminishing effects. For instance, the head left and head right positions in the prone posture were primarily determined by the head orientation and facial features. However, some important features, such as the nose, were hidden because of individual posture differences and the limited resolution of the depth camera. Similarly, there was a high variability of lower limb positions among individuals during side-lying, which challenged the classifier when distinguishing the log and fetus postures. Furthermore, the accuracy was lower in classifying the right side postures than the left side postures, which could be explained by the fixed condition sequence of the experiment. The right side postures were tested first, and the participants may have been more aware of the instruction and the requirements of the experiment. In addition, some studies reported that an individual’s dominant sleep side could contribute to accuracy bias in sleep posture classification [43,44]. A randomized cross-over trial is warranted.

We found one study that considered blankets in the sleep posture classification using a noncontact approach. Mohammad et al. [45] classified 12 kinds of sleeping postures with and without a soft blanket using a Microsoft Kinect infrared depth camera, which yielded 76% and 91% accuracy, respectively. While that system examined higher postural granularity, our classifier produced higher overall accuracy and considered more blanket conditions; in particular, the thick blanket condition was very challenging. Intriguingly, we found that the classification accuracy was greater for the thin blanket than for the no blanket. We believe that this was because the thin blanket acted as a smoothing filter to reduce noise during the classification.

Aside from the depth camera, the classification accuracy of pressure mats and wearables, such as embedded accelerometers, have been evaluated. Ostadabbas et al. [46] applied the Gaussian mixture model to process data from a pressure mat and classify three sleep postures (supine, left and right side-lying). Their system had slightly better performance (98.4%) than our system despite less granularity. In addition, Fallmann et al. [43] utilized generalized matrix learning vector quantization to classify six sleeping postures from accelerometry over the chest and both legs, with an accuracy of 98.3%. A contact-based sleep posture classification system seemed to have better performance. However, these systems can be obstructive and have maintenance issues. Wearables may be unfavorable for long-term surveillance of the elderly, since they may forget about the device after taking it off.

There were some strengths and weaknesses in our system. Our work involved a relatively larger sample size, higher granularity of posture definitions, accommodation of blanket conditions, and easy setup. Accounting for the robustness, our net was generally effective and accurate compared to the state-of-the-art methods of different modalities. However, we considered our sample to be medium sized. Our dataset also did not encompass age groups older than 70 and younger than 18. The gender ratio of the participants was also unbalanced. It is necessary to use a larger dataset in order to enhance the model’s generalizability, in particular to observe positions with finer granularity [42]. It would also be useful to construct gender subgroup models and evaluate the influence of gender on the model performance.

The long-term goal of this research is to develop a sleep surveillance system that can track participants’ sleeping postures and behaviors, and it represents a milestone in establishing baseline parameters to classify postures and remedy the pragmatic problems of blankets in a controlled setting. We achieved another milestone in monitoring the behavior of getting out of bed in a previous paper [20]. The next step is to consider real-world sleeping conditions and evaluate the proportion of each posture across the time axis.

Furthermore, sleep-associated musculoskeletal disorders and pain can manifest as significant consequences of sleep complaints and deprivation. Sleep posture itself cannot indicate whether the posture is poor or problematic, although this risk factor is modifiable and can be mitigated by using the proper mattress and pillows or engaging in physical exercise [12,47,48]. We are aiming for a machine learning-based measurement of spinal alignment and limb placement with a classification of sleeping postures that could be important in identifying related problems such as neck and back pain [12,36].

## 5. Conclusions

This study demonstrates that the overall classification accuracy of our fine-grained seven-posture system, accounting for the interference of blankets, was satisfactory at 88.9% and can pave the way toward pragmatic sleep surveillance at care homes and hospitals. We will conduct field tests on the system and enrich the dataset with participants with different health conditions. In addition, the system will be further developed to identify body morphotypes, body segment positions, and joint angles.

## Figures and Tables

**Table 1:** Overall performance of 4-posture coarse-grained and 7-posture fine-grained classification model trained by convolution neural network.

Performance	4-Posture Coarse Classification	7-Posture Fine-Grained Classification
Accuracy	97.5%	89.0%
Recall	97.3%	89.0%
Precision	97.0%	88.9%
F1 score	97.1%	88.9%
Cohen’s kappa coefficient	0.970	0.891

**Table 2:** Model performance (F1-score) of coarse- and fine-grained classifications in each posture and blanket condition.

Posture/Blanket	Thick	Medium	Thin	Control	Overall
4-posture coarse-grained classification					
Supine	92.3%	92.9%	96.0%	96.3%	94.3%
Side (right)	95.8%	98.0%	100%	98.0%	98.0%
Side (left)	98.1%	98.0%	100%	100%	99.0%
Prone	94.3%	96.2%	98.1%	100%	97.1%
Overall	95.1%	96.3%	98.5%	98.6%	97.1%
7-posture fine-grained classification					
Supine	92.9%	96.3%	96.0%	96.3%	95.3%
Log (right)	72.0%	83.3%	88.9%	87.0%	82.8%
Fetal (right)	81.5%	83.3%	84.6%	92.9%	85.7%
Log (left)	92.3%	92.9%	96.3%	96.3%	94.4%
Fetal (left)	96.0%	88.0%	91.7%	96.0%	92.9%
Prone (head right)	75.0%	78.6%	89.7%	92.9%	84.4%
Prone (head left)	81.5%	83.3%	91.7%	91.7%	86.9%
Overall	84.4%	86.5%	91.3%	93.3%	88.9%

## Data Availability

The program, model codes, and updates presented in this study are openly available in GitHub at https://github.com/BME-AI-Lab?tab=repositories (accessed on 18 July 2021). The dataset for the model is not publicly available since the videos and images of the participants would disclose their identity, violating confidentiality.

## Appendix A

The pseudocode of the algorithm is included in Table A1.

**Table A1:** The pseudocode of the model algorithm.

*0*	* Requie: a Stepsize*
*1*	$Require:\;\beta_{1},\beta_{2}$ *Exponential decay rates for the moment estimates*
*2*	$Require:\;\theta_{0}$ *: Initial parameter vector*
*3*	$m_{0}$ *← 0 (Initial 1st moment vector)*
*4*	$v_{0}$ *← 0 (Initial 2nd moment vector)*
*5*	*t ←0 : (Initial time step)*
*6*	$While\;\theta_{t}$ *not converged do*
*7*	*t ← t+1*
*8*	* Feature_vector ← BackboneNetwork(x) (Extract feature vector using CNN )*
*9*	* DropoutFeatureVector ← Dropout(Feature_vector) (Dropout layer)*
*10*	${\hat{y}}_{coarse}$*← fullyConnectedNetwork1(DropoutFeatureVector) (Predict Coarse Classification)*
*11*	${\hat{y}}_{fine}$*← fullyConnectedNetwork2(DropoutFeatureVector) (Predict Fine Classificatoin)*
*12*	$Loss\;\leftarrow CE_{coarse}\left(y_{coarse},{\hat{y}}_{coarse}\right)+CE_{fine}\left(y_{fine},{\hat{y}}_{fine}\right)$ *(Compute Loss)*
*13*	$g_{t}$ ←$\nabla_{\theta }Loss$ *(Get gradients w.r.t. stochastic objective at timestep t)*
*14*	$m_{t}$ ←$\beta_{1}*\backslash m_{t-1}+\left(1-\beta_{1}\right)*g_{t}$ *(Update biased first moment estimate)*
*15*	$v_{t}$ ←$\beta_{2}*\;v_{t-1}+\left(1-\beta_{2}\right)*g_{t}^{2}$ *(Update biased second raw moment estimate)*
*16*	$\hat{v_{t}}$←$v_{t}/\left(1-\beta_{1}^{2}\right)$ *(Compute bias-correced first moment estimate)*
*17*	$\theta_{t}$←$\theta_{t-1}-\alpha *\;\hat{m_{t}}/\left(\sqrt{\;\hat{v_{t}}}+\varepsilon \right)\;$*(Update parameters)*
*18*	*end while*
*19*	$return\;\theta_{t}$ *(Resulting parameters)*
